# Bibliometric analysis of metabolic surgery for type 2 diabetes: current status and future prospects

**DOI:** 10.1007/s13304-021-01201-5

**Published:** 2022-01-30

**Authors:** Yuling Song, Fangkun Zhao

**Affiliations:** 1 Department of Ophthalmology, The Fourth Affiliated Hospital of China Medical University, Shenyang, 110005 China; 2 Department of Endocrinology, The Fourth Affiliated Hospital of China Medical University, No. 4 Chongshan Road, Shenyang, 110032 Liaoning China

**Keywords:** Type 2 diabetes, Treatment, Metabolic surgery, Obesity, Bibliometric analysis, VOSviewer

## Abstract

Metabolic surgery has become a powerful treatment for obese type 2 diabetes (T2DM). Experts have been devoting to the research of metabolic surgery in the treatment of T2DM. The debate continues, and there is no comprehensive statistical and intuitive analysis for it. To explore the current research status, the latest hotspots and the changing trend in this field, we conducted a bibliometric analysis. This paper made a bibliometric analysis based on the data source from Science Core Collection Network (WoSCC). Vosviewer v.1.6.10 software is used to construct a knowledge map. From 2011 to 2020, 1424 peer-reviewed papers on metabolic surgery for T2DM were retrieved. The United States contributed the most publications and gained global impact with the most citations. *Obesity Surgery* was the most prolific journal in this field. Prof. Schauer Philip R., Prof. Buchwald H. and Prof. Sjostrom L. were the most renowned experts in this aspect. The top cited references discussed the status of metabolic surgery for the treatment of T2DM worldwide and the importance of regular evaluation. The extracted keywords mainly formed three clusters: (1) research on the selection of different metabolic surgery methods; (2) possible mechanisms; (3) improvement of T2DM complications by metabolic surgery. Our study makes a comprehensive and objective analysis of metabolic surgery in obese patients with T2DM, providing valuable information for further clinical application and related scientific research. Researchers can quickly locate research hotspots in a large number of relevant literature.

## Introduction

The prevalence of type 2 diabetes (T2DM) and obesity is increasing across all age groups at alarming rates [1] T2DM and obesity are closely linked to metabolic disorders, leading to increased mortality and economic burdens around the world. Especially with significant increase of cardiovascular events and cardiovascular death risks [2]. It is estimated that 80% of patients with T2DM are obese, and losing weight can actually improve obese T2DM. It was observed in the 1990s that in addition to significant weight loss, bariatric surgery also provides good blood sugar control. The Second Diabetes Surgery Summit recommended that metabolic surgery be included in glucose-lowering interventions for patients with T2DM and obesity [3]. The efficacy of metabolic surgery in the treatment of T2DM has been gradually recognized and more widely performed. Several reports have shown that metabolic surgery affects glucose metabolism independently of weight loss [4]; yet the mechanism has not been fully understood. With the increasing attention of clinicians to these research results, metabolic surgery is considered can effectively treat and prevent T2DM, and benefit patients worldwide.

Bibliometric analysis can be used to evaluate the research hotspots of a certain topic and find the direction of the field that has not been studied [5]. Researchers can choose research topics by academic evaluation of research trends. In recent years, co-citation analysis and hotspots-related keywords have been widely used in the research of knowledge-mapping analysis.

At present, thousands of original articles and comments on metabolic surgery for type 2 diabetes have been published, there is no comprehensive statistical and intuitive analysis for it. Our study bridges this gap using bibliometrics analysis and provides a comprehensive perspective on metabolic surgery for T2DM. Based on the advantages of the bibliometric analysis software Vosviewer, publications related to metabolic surgery of T2DM was statistically analyzed to reveal the core research, hotspots evolution and future development trends of related research on this topic. For this study, we assessed the annual publications growth, international collaborations, author productivity, source journals, and keyword co-occurrence analysis in relation to studies on metabolic surgery for T2DM.

## Methods

The data used for bibliometric analysis were downloaded from the Web of Science Core Collection (WoSCC) database (https://www.webofknowledge.com). We set the search criteria as follows: search topics, “metabolic surgery AND type 2 diabetes”; document type, “article”; year range, “from 2011 to 2020”; and no limit on language was set. The annual numbers of publications and the top discipline categories will be automatically generated after retrieval. VOSviewer 1.6.11, which was developed by Van Eck and Waltman of Leiden University, has the advantages of conducting accurate statistical analysis, clustering large-scale data, generating density visualization, and locating scientific research hotspots. In this study, we selected citation and co-authorship analysis to report and categorize top countries, institutions, authors, and source journals. Then, the research results are mapped from the perspective of network to illustrate the advantages of core research and reveal the collaborative relationship between them.

## Results

### Annual publications’ distribution analysis

**Fig. 1:**
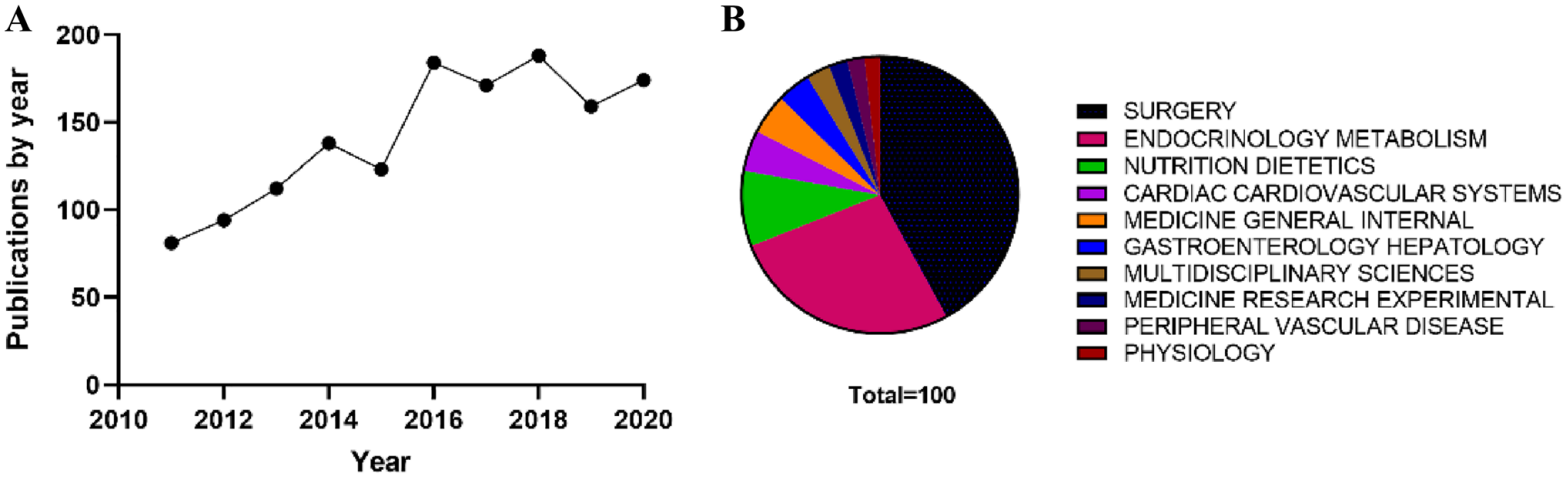
**a** The annual number of publications in metabolic surgery for T2DM research from 2011 to 2020. **b** Top 10 research areas’ distribution on metabolic surgery for T2DM

A total of 1424 articles on metabolic surgery for the treatment of T2DM were retrieved from WoSCC from 2011 to 2020 based on the bibliometric analysis results. According to statistics in Fig. 1a, the number of articles increased from 81 in 2011 to 174 in 2020. With the prevalence of obese T2DM in the world, limited drug treatment effect, the amount of metabolic surgery increased year by year. The research areas distribution is shown in Fig. 1b. Obviously, this research field is mainly distributed in Surgery (619, 43.47%), Endocrinology Metabolism (410, 28.79%), and Nutrition Dietetics (145, 10.18%), representing their center positions.

### Countries’ distribution analysis

Based on search data results, 1424 articles were provided by 63 countries. As shown in Table 1, the top 10 countries involved in T2DM metabolic surgery published 1272 articles, accounting for 89.3% of the total literature. According to citation analysis, the number of articles from the United States (392, 27.5%) is more productive, followed by China (159, 11.1%) and Italy (146, 10.2%). Meanwhile, the United States has the highest number of citations (14,253 citations, 21.4%), followed by Italy (6030 citations, 9.1%) and England (5682 citations, 8.5%). The distribution of countries could reflect the degree of connection between influential countries in this field. The more times the article was cited, the greater the influence of the country in this field.

**Table 1:** Top 10 productive countries in metabolic surgery in treatment of T2DM study, 2011–2020

Rank	Country	Count (%)	Rank	Country	Citations (%)
1	USA	392 (27.5)	1	USA	14,235 (21.4)
2	China	159 (11.1)	2	Italy	6030 (9.1)
3	Italy	146 (10.2)	3	England	5682 (8.5)
4	England	124 (8.7)	4	Australia	3488 (5.2)
5	Spain	100 (7.0)	5	China	2927 (4.4)
6	Germany	90 (6.3)	6	Germany	2881 (4.3)
7	Brazil	77 (5.4)	7	France	2462 (3.7)
8	Sweden	67 (4.7)	8	Canada	2274 (3.4)
9	France	59 (4.1)	9	Spain	2075 (3.1)
10	Canada	58 (4.0)	10	Switzerland	1893 (2.8)

Table percentages were calculated by dividing the row count by the total number of publications (*n* = 1424)

**Fig. 2:**
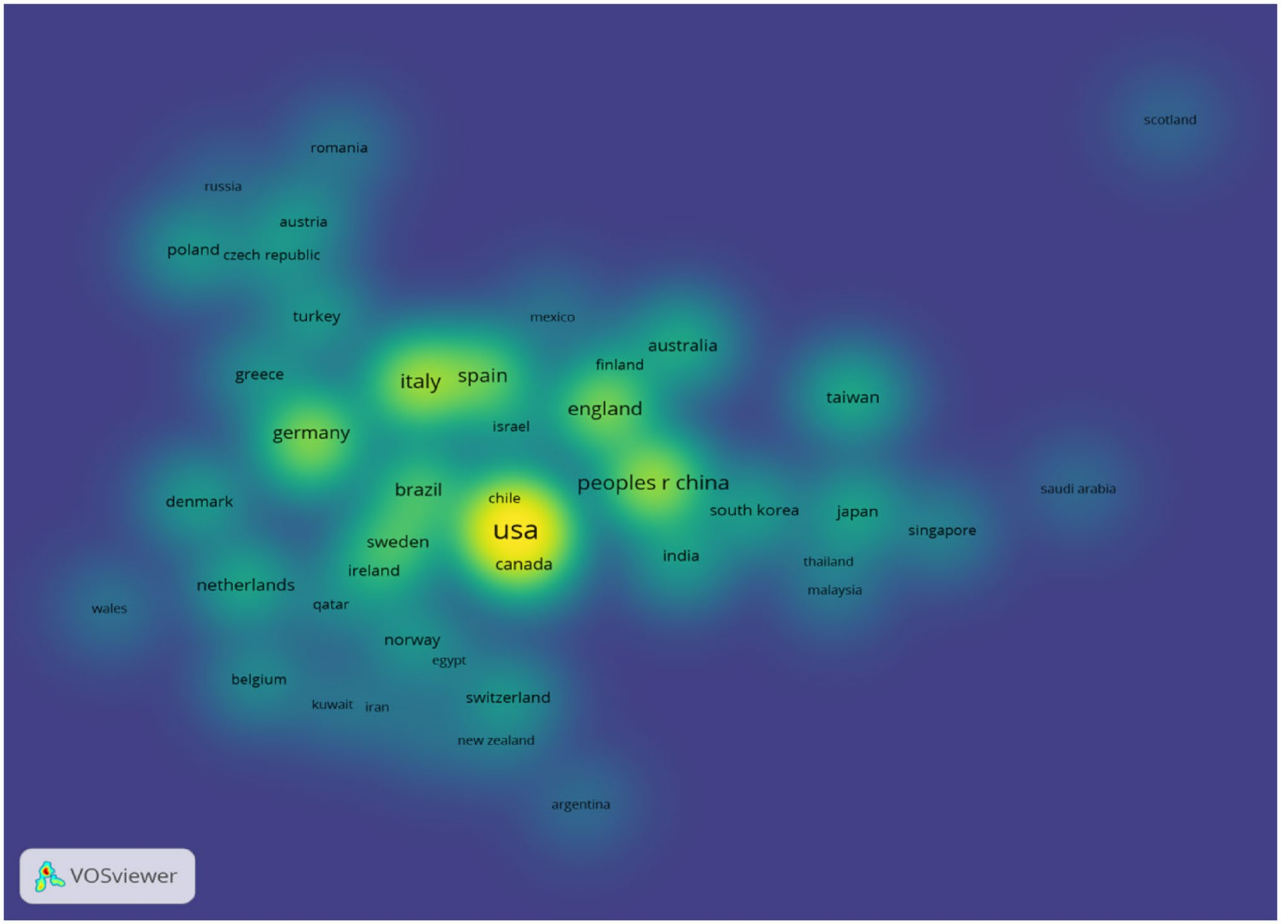
Density of main research countries in metabolic surgery in treatment of T2DM study. (The minimum number of documents of a country was set as 5. Of the 63 countries that involved in metabolic surgery in treatment of T2DM research, 47 countries meet the threshold.)

Figure 2 shows that different countries cooperate intensively, such as the United States, China, Italy, Germany, England and Canada. Cooperation between countries is expressed in terms of thickness and distance between the nodes. Besides, the size of the node represents the influence of the country. Thus, the distance between countries is not the main principal consideration affecting the partnership and more cooperation between countries could bring advanced scientific research results.

### Research organization distribution analysis

**Fig. 3:**
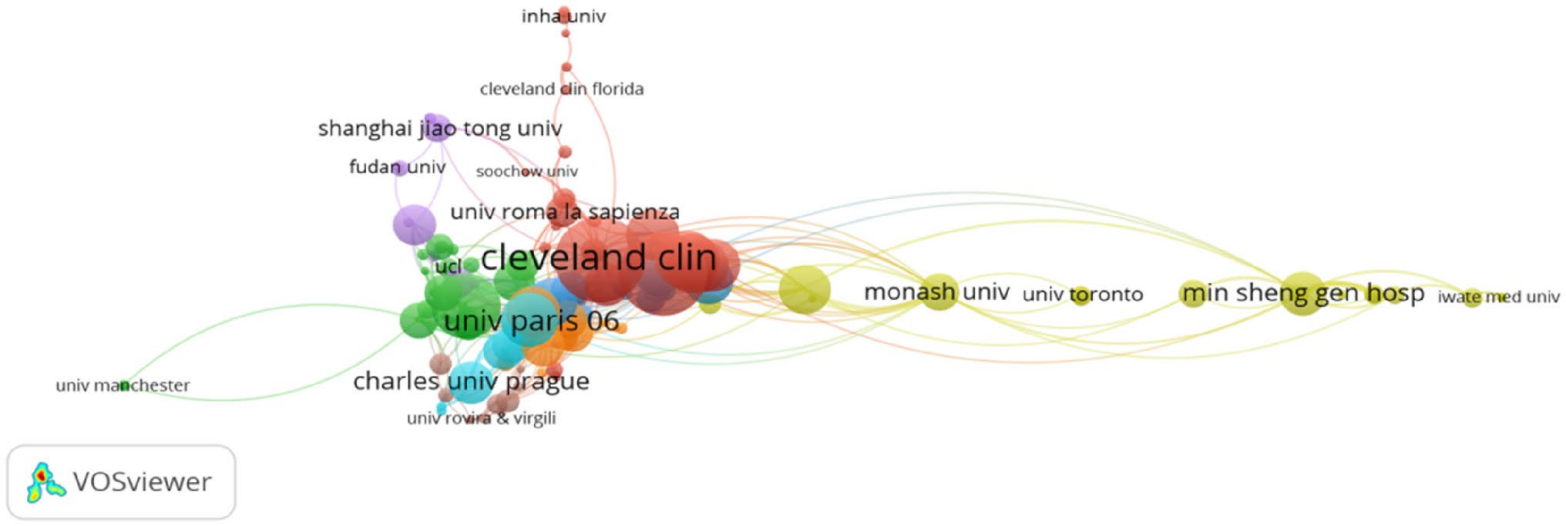
Collaboration network of main research organizations in the metabolic surgery for T2DM study. (The minimum number of documents of an organization was set as 5. Of the 1941 organizations that involved in the metabolic surgery for T2DM research, 149 organizations meet the threshold

Based on the search results, 149 organizations published 1,424 articles. As can be seen from Table 2, the top 10 organizations published 286 articles, which exceeded one-fifth of all published articles. Figure 3 shows the map of the knowledge domain of the study organization distribution in the metabolic surgery for T2DM study. The size of the node matches the number of articles retrieved. The links between nodes represent collaborations between organizations, with link strength corresponding to the intensity of collaboration.

**Table 2:** The most productive/influential organizations in the metabolic surgery for T2DM study, 2011–2020

Rank	Organization	Count (%)	Rank	Organization	Citations (%)
1	Cleveland Clinic (USA)	43 (3.3)	1	Cleveland Clinic (USA)	3122 (5.6)
2	Min Sheng General Hospital (China)	34 (2.6)	2	Harvard University (USA)	2944 (5.3)
3	Shanghai Jiaotong University (China)	32 (2.4)	3	University London Imperial College of Science, Technology and Medicine (England)	1977 (3.6)
4	University College Dublin (London)	31 (2.3)	4	University of Utah (USA)	1668 (3.0)
5	University of Gothenburg (Sweden)	30 (2.2)	5	Icahn School of Medicine at Mount Sinai (USA)	1413 (2.5)
6	College of New Rochelle (USA)	28 (2.1)	6	University Paris 06 (France)	1407 (2.5)
7	University of Sao Paulo (Brazil)	24 (1.8)	7	Mayo Clinic (USA)	1321 (2.4)
8	University of Copenhagen (Denmark)	22 (1.6)	8	College of New Rochelle (USA)	1280 (2.3)
9	Kings College London (London)	22 (1.6)	9	University of Alabama Birmingham (USA)	1278 (2.3)
10	Instituto de Salud Carlos iii (Spain)	20 (1.5)	10	Cedars Sinai Medical Center (USA)	1275 (2.2)

Percentages (%) were calculated by dividing the row count by the total number of publications (*n* = 1424)

*USA* United States of America

### Author and co-authorship distribution analysis

**Fig. 4:**
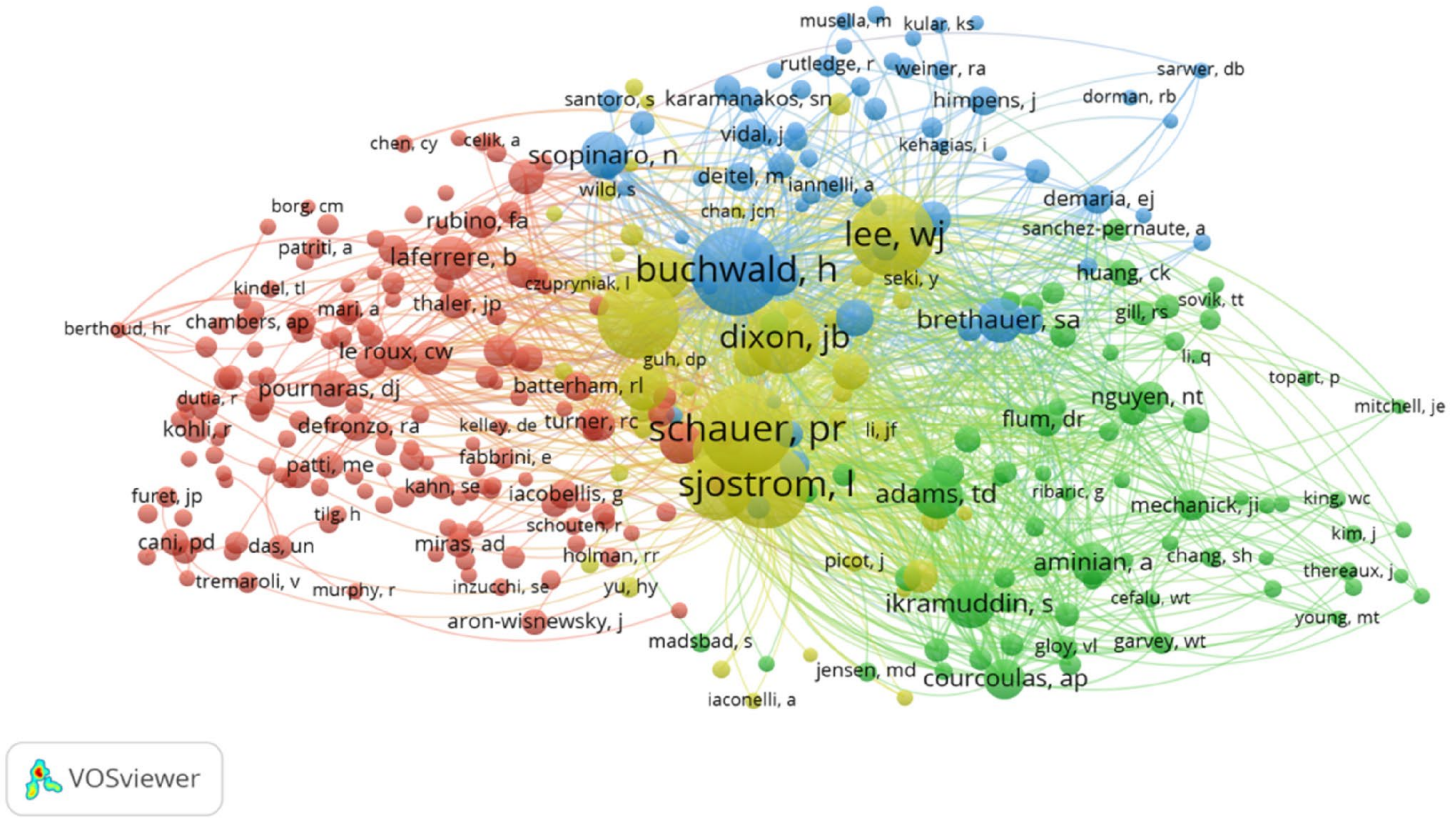
Co-authorship network of productive authors in the metabolic surgery for T2DM study. The minimum number of documents of an author was set as 20. Of the 23,422 authors that were involved in the metabolic surgery for T2DM research, 343 authors met the threshold

According to our analysis, Prof. Schauer. PR, Prof. Buchwald. H, and Prof. Sjostrom. L ranked highest among all the co-cited authors (Table 3), suggesting their influence and core status in metabolic surgery for T2DM research. Based on co-authorship analysis, Fig. 4 shows the distribution of these research groups. The citation number of publications matches the size of the nodes and the cooperation between the authors matches the degree of links between each node. The research groups were labeled with four colors, and the same group of authors was considered to be similar in their research areas and to collaborate closely with each other.

**Table 3:** Top 10 productive co-cited authors in the metabolic surgery for T2DM study, 2011–2020

Rank	Co-cited author	Citation	Institution
1	Schauer, PR	802	Cleveland Clinic (USA)
2	Buchwald, H	763	University of Minnesota (USA)
3	Sjostrom, L	705	University of Gothenburg (Sweden)
4	Lee, WJ	645	Duke University (USA)
5	Rubino, Francesco	609	Weill Cornell Medical College (USA)
6	Dixon, JB	489	Centre for Obesity Research and Education, Alfred Hospital, Melbourne (Australia)
7	Mingrone, Geltrude	427	Università Cattolica del Sacro Cuore (Italy)
8	Wslter J. Pories	243	East Carolina University (USA)
9	Adams, TD	222	University of Utah (USA)
10	Cummings, DE	221	University of Washington (USA)

### Source journal distribution analysis

Based on our search results, articles on metabolic surgery for T2DM were published in 49 journals. Table 4 provides a list of main source journals that published related topics, including the number of articles and citations in these journals. The proportion of articles published in *Obesity Surgery* and *Surgery For Obesity and Related Diseases* accounted for 31.9% of all publications in this study, and ranked the top two in citations as well (39.5%).

**Table 4:** Top 10 main source journals in the metabolic surgery for T2DM study, 2011–2020

Rank	Journal	Count (%)	Rank	Journal	Citations (%)
1	Obesity Surgery (USA)	232 (16.3)	1	Obesity Surgery (USA)	4970 (20.7)
2	Surgery for Obesity and Related Diseases (Netherlands)	223 (15.6)	2	Surgery for Obesity and Related Diseases (Netherlands)	2523 (18.8)
3	Nutrition Metabolism and Cardiovascular Diseases (Netherlands)	53 (3.7)	3	Diabetes Care (USA)	2809 (11.7)
4	Diabetes Care (USA)	37 (2.6)	4	Lancet (England)	1107 (4.6)
5	Plos One (USA)	25 (1.7)	5	Annals of Surgery (USA)	1105 (4.6)
6	Journal of Clinical Endocrinology Metabolism (USA)	21 (1.5)	6	Gut (England)	1045 (4.3)
7	Surgical Endoscopy and Other International Techniques (USA)	20 (1.4)	7	Obesity (USA)	847 (3.5)
8	International Journal of Obesity (England)	19 (1.3)	8	Journal of Clinical Endocrinology Metabolism (USA)	692 (2.9)
9	Diabetologia (Germany)	18 (1.2)	9	Diabetologia (Germany)	648 (2.7)
10	Annals of Surgery (USA)	15 (1.1)	10	Obesity Facts (Switzerland)	575 (2.4)

### Keywords’ analysis

**Fig. 5:**
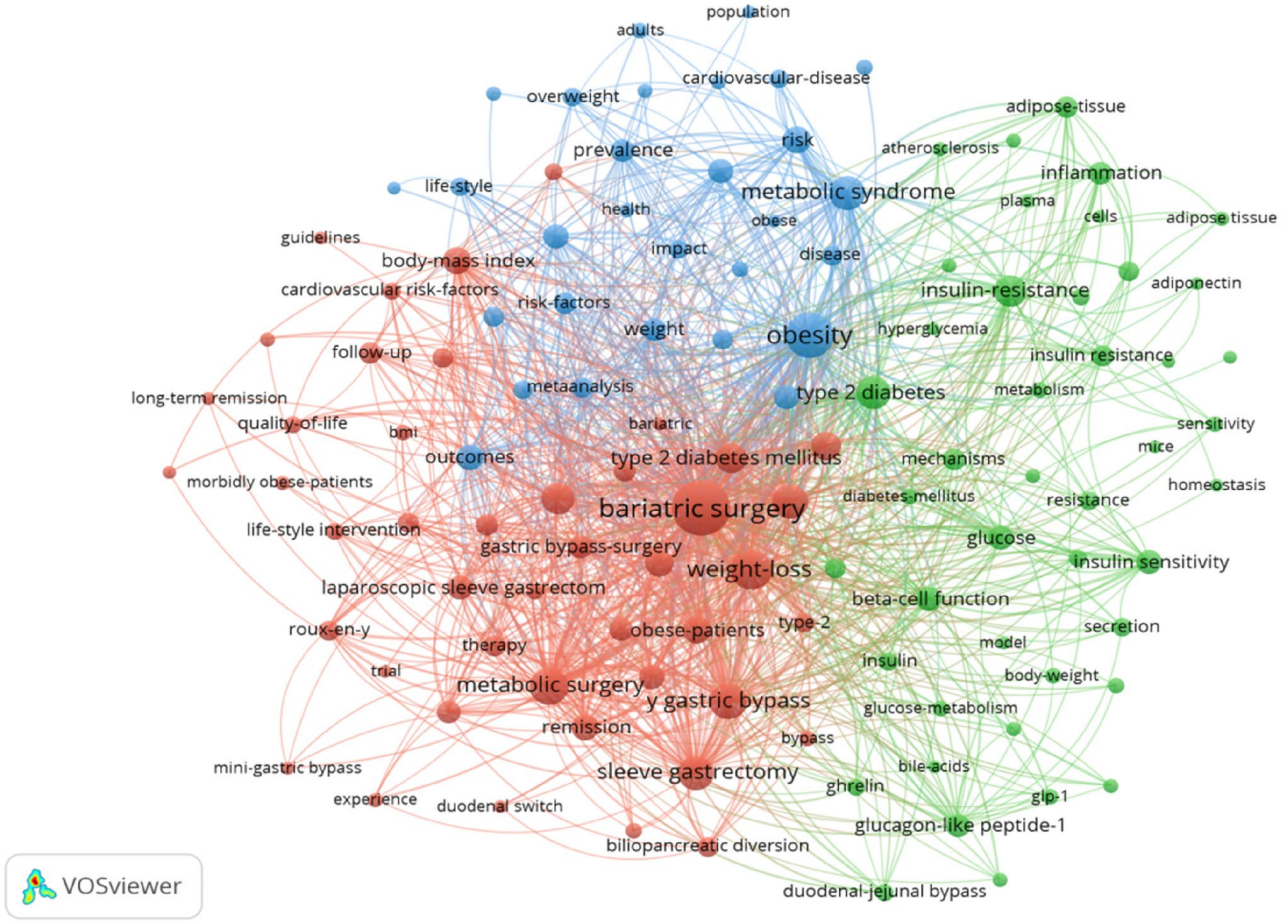
Co-occurrence network of keywords in the metabolic surgery for T2DM study. (The minimum number of occurrences of a keyword was set as 20. Of the 4185 keywords that involved in the metabolic surgery for T2DM research, 118 keywords meet the threshold.)

The research hotspots for treatment of metabolic surgery for T2DM were identified by high-frequency keyword co-occurrence analysis. The minimum co-occurrence of the keyword was set to 20. Of the 4185 keywords extracted for metabolic surgery for T2DM, 118 keywords reached the threshold, and those keywords with similarities were clustered based on the network. The three main clusters were represented by red, green, and blue, respectively (Fig. 5). Each cluster lists its top ten keywords (Table 5). Table 6 lists the top ten cited articles of the last decade on the basis of WoSCC. The keywords clusters represent new advances in this field over the past decade, and help readers figure out the hot spots more effectively. In addition, these ten articles, in turn, can be used to explain the distribution of keywords according to their contents.

**Table 5:** Co-occurrence analysis of keywords

Cluster 1 (red)	Cluster 2 (green)	Cluster 3 (blue)
Bariatric surgery (869)	Type 2 diabetes (209)	Obesity (508)
Weight-loss (383)	Insulin-resistance (204)	Metabolic syndrome (240)
Metabolic surgery (308)	Glucose (103)	Risk (128)
Y gastric bypass (272)	Insulin sensitivity (102)	Outcomes (118)
Sleeve gastrectomy (258)	Beta-cell function (100)	Association (103)
Gastric bypass (236)	Glucagon-like peptide-1 (92)	Mortality (101)
Type-2 diabetes-mellitus (198)	Inflammation (89)	Surgery (96)
Body-mass index (138)	Insulin resistance (78)	Prevalence (89)
Obese-patients (135)	Adipose-tissue (75)	Weight (83)
Remission (120)	Mechanisms (73)	Risk-factors (79)

**Table 6:** Top 10 citation reports in the metabolic surgery for T2DM research, 2011–2020

Rank	Title (citation times)	Keywords	Cluster
1	Metabolic/bariatric surgery worldwide 2011. PMID: 15915461 (874)	Bariatric surgery	1
2	Clinical practice guidelines for the perioperative nutritional, metabolic, and nonsurgical support of the bariatric surgery patient-2013 update: cosponsored by American Association of Clinical Endocrinologists, The Obesity Society, and American Society for Metabolic and Bariatric Surgery. PMID: 9547102 (602)	Metabolic surgery	1
3	Akkermansia muciniphila and improved metabolic health during a dietary intervention in obesity: relationship with gut microbiome richness and ecology. PMID: 26707365 (596)	Mechanisms	2
4	Bariatric surgery versus non-surgical treatment for obesity: a systematic review and meta-analysis of randomised controlled trials. PMID: 28241825 (568)	Outcomes	3
5	Bariatric-metabolic surgery versus conventional medical treatment in obese patients with type 2 diabetes: 5 year follow-up of an open-label, single-centre, randomised controlled trial. PMID: 20427778 (566)	Outcomes	3
6	Severe obesity in children and adolescents: identification, associated health risks, and treatment approaches a scientific statement from the American Heart Association. PMID: 22488764 (485)	Risk-factors	3
7	AMPK, insulin resistance, and the metabolic syndrome. PMID: 21038418 (460)	Mechanism	2
8	Management of type 2 diabetes: new and future developments in treatment. PMID: 21623852 (352)	Mechanism	2
9	Clinical Practice guidelines for the perioperative nutritional, metabolic, and nonsurgical support of the bariatric surgery patient-2013 update: cosponsored by American Association of Clinical Endocrinologists, The Obesity Society, and American Society for Metabolic and Bariatric Surgery. PMID: 16227287 (351)	Metabolic surgery	1
10	Metabolic surgery in the treatment algorithm for type 2 diabetes: a joint statement by International Diabetes Organizations. PMID: 15565570 (350)	Outcomes	3

## Discussion

### Global trends

After decades of development, a large number of clinical trials have confirmed that metabolic surgery is a practical and effective treatment method to correct metabolic disorders in addition to weight loss, especially in cases where drug treatment is difficult to maintain long-term stable efficacy. Metabolic surgery for obese T2DM has become the consensus of experts in the new stage of diabetes treatment. As shown in Fig. 1, the publication number arises from 81 in 2011 to 174 in 2020. Increasing patient awareness of the impact of diabetes and overweight on adverse cardiovascular outcomes, improved surgeon skills, and the inclusion of metabolic surgery in insurance coverage are reasons for the increasing number of metabolic surgeries performed each year. There is an overall growth in the volume of literature, however, the rate of increase is slow. This may be because long-term clinical trials tend to require longer cycles to assess the efficacy and risk of the surgeries, thereby clarifying hard endpoints such as postoperative cardiovascular outcomes.

According to country distribution analysis (Fig. 2), eight of the top ten countries in terms of publication volume are developed countries and two are developing countries. In the research on weight loss by metabolic surgery, publications in developed countries are mainly related to the high incidence of obesity, dietary structure and lifestyle, whereas publications in developing countries, including China and Brazil, are mainly associated with urbanization and a high prevalence of obesity. Influenced by race and diet structure, more than 40% of American diabetic patients have a body mass index (BMI) over 30 kg/m^2^. Although the majority of Asian diabetic patients have BMI between 27 and 30 kg/m^2^, [1] they can also benefit from metabolic surgery metabolic surgery. Cooperation between different countries will help to explore the indications of metabolic surgery, so as to gain worldwide endorsement through high degree of vetting.

Through citation analysis, we can retrieve publications which have been cited more frequently, so as to provide us a quick insight into the research of this field. Approximately 30% of adults in the United States are obese [6]. Metabolic surgery increases with the incidence of obesity, resulting in accumulation of more clinical experience and increased citation of publications. As we can see from Table 1, publications of the US ranked first with 14,235 citations.

By analyzing the distribution of research organizations, we can identify the most productive organizations in a certain field and cooperation within the group. As shown in Table 2, the top three influential institutions are Cleveland Clinic, Harvard University and University London Imperial College of Science, Technology and Medicine, which hold the core positions of research organizations in the entire research network. Through authorship analysis, relevant researchers can look for possible cooperation opportunities through the establishment of a network knowledge map of coauthors. The number of citations reflects the influence of these authors in this field. The top ten productive co-cited authors in treatment of metabolic surgery for T2DM study from 2011 to 2020 are listed in Table 3, 70% of whom are from the United States. They are recognized as experts in this field. Perhaps their common research goals and geographical proximity enabled them to cooperate in the same field.

Distribution analysis of academic journals can help researchers understand the core journals in this field and assist in journal selections. As shown in Table 4, “Obesity Surgery” was the most cited journal with 4,970 citations. Based on our findings, “Obesity Surgery” has published the most papers in metabolic surgery for T2DM research and is considered the most prolific journal in this field with a wide influence.

A great quantity of cited reference documents can show the background with advantage through co-citation analysis. Consequently, we performed a cluster analysis on the main topics of metabolic surgery for T2DM.

### Research frontiers

The co-occurrence of keywords could reflect the search topic and reveal the internal structure of references and frontier disciplines. As shown in Fig. 5, three clusters are formed in the theme of metabolic surgery for T2DM, and similar keywords in the research theme are combined together. We combined the characteristics and current status of metabolic surgery for T2DM research to analyze the formation of these three clusters.

Cluster 1 (red colored) includes keywords representing different metabolic surgical procedures. As shown in Table 5, the top keywords of this cluster are “y gastric bypass”, “sleeve gastrectomy” and “gastric bypass”. Henry Buchwald et al. [7] reported 78.1% of patients with diabetes had complete resolution after metabolic surgery, and 86.6% of them were improved or resolved (co-citation no. 1). Despite the high short-term success rate of metabolic surgery, there are still some diabetic patients who fail to achieve continuous control of blood glucose after surgery. There are limited data on the long-term outcomes and durability of metabolic surgery for obese T2DM. According to our citation reports, the article discussed about “periodic assessment of the state of metabolic surgery” [8] ranked first (Table 6) (citation no. 1). Clinicians are always eager to simplify procedures and minimize their side effects and complications, thus, with the emergence of new types of surgeries, old ones fell into disuse. In the development of metabolic surgery for T2DM, many surgical methods have been recommended for weight loss [9]. Most of them were abandoned because of serious complications, while some were abandoned because of difficulty, time-consuming and lack of experience. Those surgeries that can obtain sustained weight loss and blood glucose control, as well as fewer surgical complications, are undoubtedly recognized by experts. Through periodic assessment of the state of metabolic surgery, comparison of various surgical procedures for the treatment of diabetes, surgical complications, safety and the influence of cardiovascular disease, we can provide more theoretical basis for clinicians. The success of operation is also inseparable from perioperative nutritional support. Jeffrey I. Mechanick et al. [10] published clinical practice guidelines on nutritional, metabolism, and nonsurgical support in the perioperative period of metabolic surgery (citation no. 2). This article ranks second among the top 10 citations, indicating that these guidelines for successful operation are important.

Researchers have tried to explore the best biological and clinical predictors of postoperative remission of T2DM, including BMI, age, islet function, preoperative application of hypoglycemic drugs and surgical types et al. Some of the studies are case study and some are retrospective study and cohort study. Geltrude Mingrone et al. [11] considered that preoperative BMI and weight loss did not predict improvement in postoperative hyperglycemia (co-citation no. 4). However, some prospective studies have shown that age and insulin therapy are associated with lower long-term success rate [9], indicating the potential benefits of early intervention. The inconsistencies maybe because of lacking large RCT studies to further clarify how to better improve the prognosis/outcome of metabolic surgery and to make the curative effect more durable.

If we can elucidate the predictors of long-term remission of T2DM and determine which groups of people respond poorly to metabolic surgery, we can inform patients preoperatively of the likelihood of a suboptimal outcome. Doctors can be more specific for the surgical population, and patients can make more rational decisions about surgery.

The development of minimally invasive laparoscopic techniques has led to an overall increase in the safety of metabolic surgery. It also reduces the risk of operative mortality. However, the side effects such as trace element deficiency and anemia cannot be avoided. In addition, there is a certain contradictory relationship between weight loss and cardiovascular disease after metabolic surgery. Some studies shown that trimethylamine nitric oxide (TMAO) increases after RyGB both in animal and human [12, 13]. However, plasma TMAO concentration is exactly a predictor of atherosclerosis and cardiovascular events [14, 15]. Obviously, the conclusions are inconsistent, and it is necessary to further explore the surgical indications and mechanisms. Identifying the mechanism is critical to further exploring treatments that could cure obese T2DM without the need for metabolic surgery.

Cluster 2 (green colored) represents keywords related to the mechanism of metabolic surgery for T2DM. As shown in Table 5, the top keywords of this cluster are “insulin -resistance”, “insulin sensitivity”, “beta-cell function”, “glucagon-like peptide-1”, and “inflammation”. According to our citation reports, citations corresponding to keywords in cluster 2 are No.3, No.7, and No.8 (Table 6). The possible mechanisms of T2DM remission include: incretin effect, bile acid signaling, gastric volume reduction, nutritional changes after anatomical remodeling, changes in vagus and endocrine hormones, circulating branched-chain amino acids, and intestinal flora [1]. Among them, the role of incretin has received the most attention, especially glucagon-like peptide 1 (GLP-1) and gastric inhibitory polypeptide (GIP). Gastric bypass surgery has been reported to increase GLP-1 and YY peptide levels while decreasing basal ghrelin levels (citation no. 8) [16]. GLP-1 receptor agonist is currently a commonly used hypoglycemic drug in the treatment of obese T2DM. It is the first choice for obese T2DM with high risk factors of cardiovascular disease. Typical representative drug is liraglutide, which has been approved in the US and other countries for weight loss treatment of non-diabetic obese people. However, drug therapy had limited effect on weight loss.

Studies have shown that metabolic surgery has a profound impact on intestinal microflora. Maria Carlota Dao et al. 20 (citation no. 3) reported that Akkermansia muciniphila is a kind of mucin-degrading bacteria. Akkermansia muciniphila increases with bariatric surgery in both humans and mice, and is thought to help reduce body fat mass, glucose homeostasis, reduce adipose tissue inflammation, and increase intestinal integrity. The gut bacteria could be considered therapeutic targets. Therefore, further investigation may make it a therapeutic option, for example, to select suitable bacteria for intestinal flora transplantation.

The mechanism of AMP-activated protein kinase (AMPK) has also attracted extensive attention. Neil B. Ruderman et al. [17] (citation no .7) reported AMPK has been linked to insulin resistance. The activity of AMPK in adipose tissue decreased significantly after operation. Identification of effective and specific AMPK activators may be a potential nonsurgical treatment for obese T2DM someday.

Cluster 3 (blue colored) represents keywords related to outcomes, association, risk factors, mortality of metabolic surgery for T2DM research (Table 6). According to our citation reports, citations corresponding to keywords in cluster 3 are citation no. 4, no. 5, no. 6, and no. 10.

Sjostrom et al. [18] (co-citation no. 7) have long established that bariatric surgery for severe obesity is associated with long-term weight loss and lower overall mortality. Moreover, many scholars compared the effect of operation and conventional drugs on obesity T2DM [11, 19] (co-citation no. 4 and 5). The outcomes showed that metabolic surgery is more effective than conventional drugs in the treatment of these patients. It can significantly reduce diabetes-related complications and the use of glucose-lowering drugs, and is more effective in reducing cardiovascular risk and mortality. Furthermore, it has been reported that the combination of internal and external medicine is more effective than direct surgery, which is more conducive to the remission of diabetes. Schauer et al. [20] (co-citation no. 8) reported 3-year outcomes, that intensive medical therapy plus bariatric surgery received better results than surgical therapy alone. Francesco Rubino et al. [3] (citation no. 10) considered that the success of metabolic surgery needs to be determined in the context of a comprehensive diabetes treatment plan. It seems that the multi-disciplinary treatment with the participation of metabolic medicine, nutrition, basic medicine and cardiology experts may be the development trend in the future.

According to our results, most authors focused on the study of metabolic surgery and macroangiopathy. Studies on metabolic surgery and microvascular lesions are limited. Buchwald et al. [21] (co-citation no. 2) shows a systematically reviewed and subjected to meta-analysis outcome. It is reported that the vast majority of obese patients with metabolic diseases such as diabetes achieve complete remission or improvement through metabolic surgery. Schauer et al. [22] reported that (co-citation no. 3) 12 months of medical therapy combined with metabolic surgery resulted in significantly more patients with glucose control than drug therapy alone. The curative effect is mainly reflected in the improvement of HbA1c, blood lipid and complications, and good cardiovascular outcome. Although data are limited, there have been studies of microvascular lesions. Chen et al. [23] reported that bariatric surgery could not prevent the progression of DR, while Kim [24] and Merlotti [25] reported positive results. The inconsistent results may be related to sample size and follow-up time. More randomized clinical trials are needed to confirm the effect of metabolic surgery on the hard end points of diabetic microvascular complications.

Obesity on cardiovascular effects not limited to adults, also affects children and adolescents. Kelly et al. [26] reported that (citation no. 6) adolescents with high levels of obesity are more likely to have an aggregation of cardiovascular disease risk factors. But due to the lack of long-term results, these population do not have the operation qualification surgical indications, the lack of insurance coverage, the metabolism of weight loss surgery in the application of these people is limited. They have to resort to drugs, exercise and lifestyle improvements. The discussion mainly focused on the indication of operation and extreme weight. More evidence-based medical evidence and scientific clinical trials may broaden the indications for surgery in this population and bring them hope of treatment.

Despite these advantages, some limitations should also be considered. First, papers extracted from WoSCC from 2011 to 2020 may not cover all topics of metabolic surgery in T2DM research. WoSCC is a more suitable database for citation analysis. Second, most of the publications of WoSCC are in English, which may lead to linguistic bias. In addition, cooperative network analysis can show the co-occurrence and co-authorship of the institution, but VOSviewer cannot generate geographic map of co-author in the output file. Therefore, it is not possible to generate the visualization of geographical location and co-author, nor to calculate an understanding of their relationships.

In conclusion, through the bibliometric analysis and visualization method, the current study provides a new perspective to analyze the application of metabolic surgery in the treatment of T2DM. This metabolic surgery treatment is a promising direction globally, with great potential for improving T2DM therapies. Our study makes a comprehensive and objective analysis of metabolic surgery in obese patients with T2DM. We expect this work may provide valuable information for further clinical application and related scientific research.
